# Polypharmacy prevalence in older adults seen in United States physician offices from 2009 to 2016

**DOI:** 10.1371/journal.pone.0255642

**Published:** 2021-08-03

**Authors:** Eric H. Young, Samantha Pan, Alex G. Yap, Kelly R. Reveles, Kajal Bhakta

**Affiliations:** 1 College of Pharmacy, The University of Texas at Austin, Austin, Texas, United States of America; 2 Pharmacotherapy Education & Research Center, UT Health San Antonio, San Antonio, Texas, United States of America; University of South Australia, AUSTRALIA

## Abstract

**Background/objectives:**

With an aging population suffering from increased prevalence of chronic conditions in the United States (U.S.), a large portion of these patients are on multiple medications. High-risk medications can increase the risk for drug-drug interactions and medication nonadherence. This study aims to describe the prevalence of polypharmacy and high-risk medication prescribing in U.S. physician offices.

**Methods:**

This was a cross-sectional study of the Centers for Disease Control and Prevention’s National Ambulatory Medical Care Survey from 2009 to 2016. All patients over 65 years old were included. Polypharmacy was categorized as no polypharmacy (< 2 medications), minor polypharmacy (2–3 medications), moderate polypharmacy (4–5 medications), and major polypharmacy (>5 medications). Medications were further categorized into high-risk medication categories (anticholinergics, cardiovascular agents, central nervous system (CNS) medications, pain medications, and other). Comparisons between the degrees of polypharmacy were performed utilizing chi-square or Wilcoxon rank-sum tests with JMP Pro 14^®^ (SAS Institute, Cary, NC).

**Results:**

Over 2 billion patient visits were included. Overall, Polypharmacy was common (65.1%): minor polypharmacy (16.2%), moderate polypharmacy (12.1%), and major polypharmacy (36.8%). Patients with major polypharmacy were older compared to those with moderate or minor polypharmacy (75 vs. 73 years, respectively) and were most frequently prescribed pain medications (477.3 per 1,000 total visits). NSAIDs were the most frequently prescribed, with 232.4 per 1,000 total visits resulting in one high-risk NSAID prescription, while 21.9 per 1,000 total visits resulted in two or more high-risk NSAIDs.

**Conclusion:**

Most patients over 65 years experienced some degree of polypharmacy, with many experiencing major polypharmacy. This indicates an increased need for expanded pharmacist roles through medication therapy management and safety monitoring in this patient population.

## Introduction

Unhealthy aging is a growing concern in the United States (U.S.), particularly in the older population. Over 13% of the U.S. population is comprised of adults 65 years and older, and this number continues to increase [1]. As the population in the U.S. begins to age, there is an increasing risk of patients experiencing multiple chronic conditions [2]. This may result in a greater prevalence of polypharmacy, which is the concomitant use of multiple medications where their therapeutic benefits may be outweighed by harm, such as increased adverse events and poor health outcomes [2, 3]. This gradual change has been observed over the past several years, especially in older individuals [4].

An aging population combined with polypharmacy may lead to the prescribing of potentially inappropriate medications (PIMs), which can not only be ineffective for the patient’s condition, but can also lead to a higher risk for developing adverse drug events (ADEs), including drug-drug interactions (DDI) and drug-disease interactions, as well as medication non-adherence due to increased pill burden [3]. These factors can ultimately result in negative health implications, including a decline in functional and cognitive status [2]. As about half of the ADE-related hospital admissions are preventable, the presence of ADEs may consequently lead to increased hospitalizations, which can be due to gastrointestinal (GI) bleeding, anemia associated with bleeding, hypotension, and syncope, among others [5–7]. Development of these ADEs may prompt additional medication intervention to treat those issues, further exacerbating medication use and burden on patients.

Older adults with polypharmacy have also been found to be at a higher risk of experiencing DDIs or drug-disease interactions [8]. In a prospective study, Doan et al. found that 80% of older hospitalized patients with polypharmacy had potential cytochrome (CYP) P450-mediated DDIs. The probability of having at least one CYP-mediated DDI increased with the number of medications. For example, in patients aged 66 years and older who were admitted to the hospital for a drug-related toxicity, those diagnosed with hypoglycemia, digoxin-toxicity, and hyperkalemia were 6, 12, and 20 times more likely to have taken an interacting medication the previous week, respectively [8]. Other medications with similar indications and no known interactions were not found to increase the risk of drug toxicity [9].

Due to the complexities of polypharmacy, the role of medication education and safety have greatly expanded throughout all healthcare professions, ranging from improved medication adherence in nurse-led interventions to medication therapy management (MTM) conducted by pharmacists [10]. In addition, guidelines like The American Geriatrics Society (AGS) Beers Criteria^®^ for Potentially Inappropriate Medication Use in Older Adults have been published to promote the safe use and prescribing of these medications to prevent harmful effects. Beer’s Criteria is a comprehensive evidence-based tool that outlines inappropriate medication use in vulnerable populations. Based on thousands of clinical trials and studies, it includes recommendations for alternative medications to use as well as adjusted dosing schedules and common drug-drug interactions to avoid [11]. Other criteria, like the Screening Tool of Older Person’s Prescriptions (STOPP), and the Screening Tool to Alert to Right Treatment (START), have also been used by physicians and pharmacists to improve clinical outcomes in older patients with numerous chronic conditions through medication intervention [12].

While these tools exist to aid in safe medication prescribing, it is imperative to evaluate how frequently these high-risk medications are prescribed in the older population, particularly in those on multiple medications. Promoting judicious medication prescribing for this patient population may provide benefit for not only adequately treating these chronic conditions, but also helping to reduce ADEs and unnecessary pill burden [13]. Therefore, this study aims to quantify the prevalence of polypharmacy and to determine the rates of high-risk medication prescribing in older U.S. outpatients.

## Methods

### Data source and ethics statement

This was a cross-sectional study using data from the CDC’s National Ambulatory Medical Care Survey (NAMCS) from 2009 to 2016. The NAMCS utilizes a sample of visits within non-federal employed office-based outpatient physicians (e.g., private practice and free-standing clinics) in the U.S. Providers in this survey are assigned to a one-week reporting period, where information is collected on patient demographics, diagnoses, medications ordered or provided, any diagnostic procedures performed, and future treatment plans. From 2009 to 2013, the database contains a total of three diagnoses and 8 to 10 medications collected for each patient visit. From 2014 to 2016, these were expanded to five diagnoses and 30 medications.

The UT Health Science Center San Antonio Institutional Review Board waived formal ethics approval and patient consent, as these data are publicly available and do not contain any patient identifiers.

### Patient population and study definitions

Patients that were 65 years and older during their visit in the NAMCS from 2009 to 2016 were included in this study. Patient visits were further categorized by overall visits and degree of polypharmacy, which was defined by the number of medications that were ordered or documented at the time of their visit. Although polypharmacy can be defined multiple different ways, the authors chose to classify polypharmacy with the following method to adjust for the degree of polypharmacy: no polypharmacy (less than 2 medications), minor polypharmacy (2 to 3 medications), moderate polypharmacy (4 to 5 medications), and major polypharmacy (more than 5 medications). Furthermore, inappropriate or high-risk medications were identified in this study. High-risk medications were defined by their respective Multum code, a six-digit generic-equivalent code that reflects up to six components that make up a given drug (S1 Table). Combination drugs were also reflected by their respective Multum code, although potentially counted as multiple drugs based on their therapeutic ingredients. These medications were further classified based on The AGS Beers Criteria^®^ for Potentially Inappropriate Medication Use in Older Adults, where medications that belong to this list are recommended to be avoided by older adults in a majority of circumstances, including specific diseases or conditions [11]. These medications were further broken down into five major medication categories: anticholinergics, cardiovascular agents, central nervous system (CNS) medications, pain medications, and other (endocrine medications, gastrointestinal agents, and anti-infectives). A full table of these medication categories can be seen in S1 Table. Lastly, medication classes of interest were categorized within each medication category.

### Data and statistical analyses

Descriptive and statistical data analyses were conducted using JMP Pro 14^®^ (SAS Institute, Cary, NC). Data weights provided by the NAMCS were used to extrapolate sample visits to national estimates and were used for all analyses in this study. Baseline characteristics were compared between individuals experiencing varying degrees of polypharmacy (minor polypharmacy, moderate polypharmacy, and major polypharmacy) and those with no polypharmacy utilizing chi-square or Wilcoxon rank-sum tests where appropriate. This study also analyzed the rates of high-risk medication prescribing, which were calculated as the number of visits that included a high-risk medication class per 1,000 total outpatient visits. Lastly, a subgroup analysis of the most common chronic conditions seen by high-risk medication class was performed on patients who experienced major polypharmacy.

## Results

### Population characteristics

A total of 2.1 billion visits with an overall median number of 3 (IQR 1–8) medications were included for analysis. Of those visits, a total of 1.4 billion visits (65.1%) had some form of polypharmacy, with 738,923,785 (34.9%) including patient visits with no polypharmacy, 341,779,401 (16.2%) with minor polypharmacy, 256,247,011 (12.1%) with moderate polypharmacy, and 779,309,084 (36.8%) with major polypharmacy. Baseline characteristics are presented in Table 1. Overall, patients with major polypharmacy were older compared to those with moderate or minor polypharmacy (75 vs. 73 years, respectively), while patients with minor or moderate polypharmacy were younger than those with no polypharmacy (73 vs. 74 years, respectively). Sex, race, and ethnicity were all numerically similar between groups, but were statistically different because of a large sample size. Lastly, patients with major polypharmacy had a higher median number of total chronic conditions (3), compared to those seen with no polypharmacy (0), minor polypharmacy (2), and moderate polypharmacy (2).

**Table 1:** Baseline characteristics by degree of polypharmacy.

Characteristic^+^	No polypharmacy (n = 738,923,785)	Minor polypharmacy (n = 341,779,401)	Moderate polypharmacy (n = 256,247,011)	Major polypharmacy (n = 779,309,084)
**Age (years), median (IQR)**	74 (69–80)	73 (68–79)	73 (68–80)	75 (70–81)
**Female sex, %**	55.5	57.5	56.1	57.5
**Race, %**				
White	66.2	62.5	66.0	67.2
Black/African American	5.7	6.0	7.2	6.4
Other	4.3	4.7	4.0	2.9
More than one race	0.1	0.1	0.2	0.2
**Hispanic or Latino ethnicity, %**	7.1	7.7	7.9	6.1
**Chronic conditions, median (IQR)**	0 (1–2)	2 (1–3)	2 (1–3)	3 (2–4)

^+^ All comparisons between no polypharmacy vs. minor polypharmacy vs. moderate polypharmacy vs. major polypharmacy groups were deemed statistically significant if p<0.0001

### Rates of high-risk medication prescribing overall and by degree of polypharmacy

**Fig 1:**
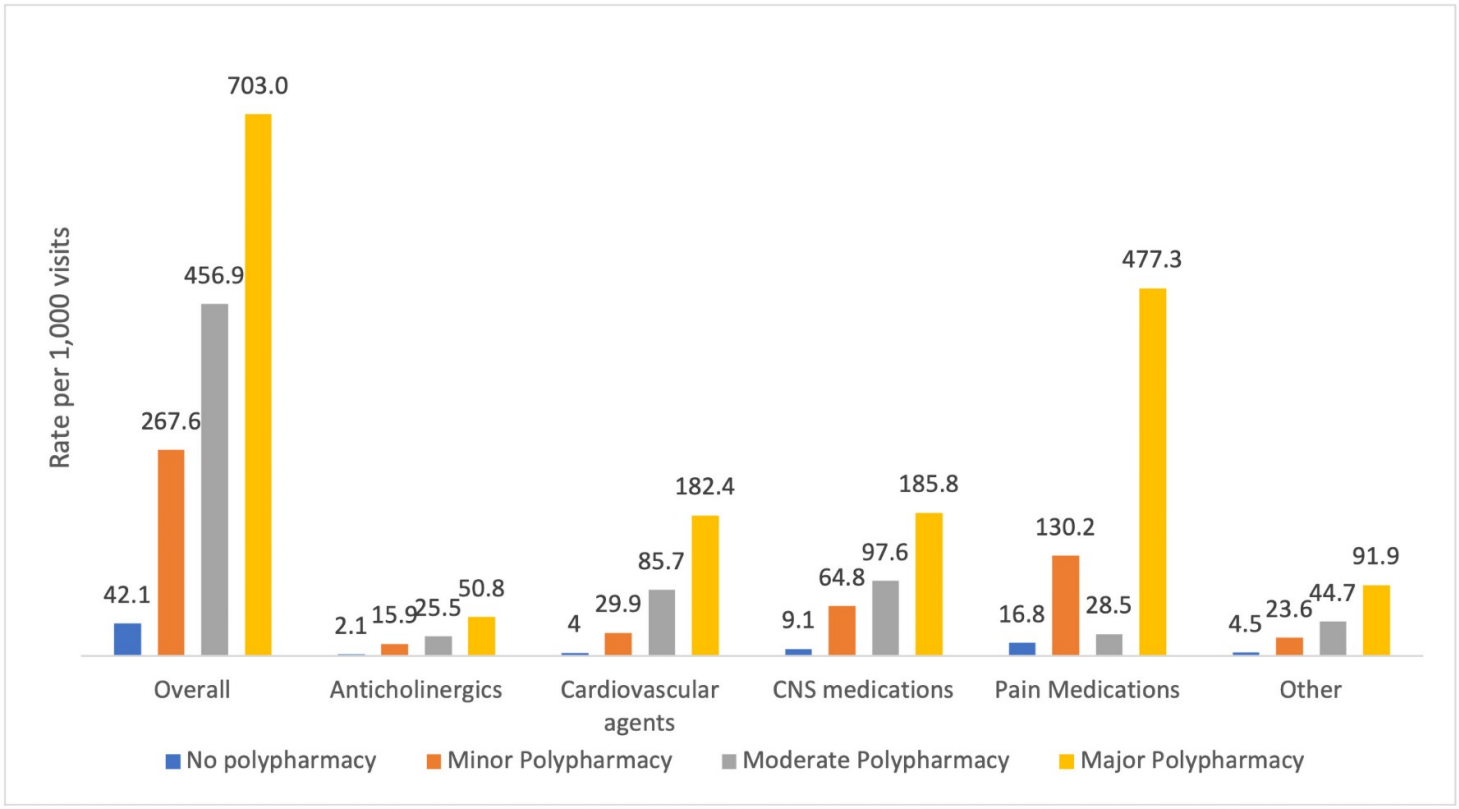
High-risk medication prescription rates by medication category. ^+^ All comparisons between no polypharmacy vs. minor polypharmacy vs. moderate polypharmacy vs. major polypharmacy groups were deemed statistically significant (p<0.0001).

Patients who exhibited a higher degree of polypharmacy (i.e., major polypharmacy) had a documented high-risk medication prescribed compared to those exhibiting a lesser degree of polypharmacy (i.e., minor and moderate polypharmacy). Overall, 703.0 per 1,000 patient rate visits with major polypharmacy had a documented high-risk medication prescribed. This rate declined with less severe forms of polypharmacy (moderate polypharmacy, 456.9 per 1,000, minor polypharmacy, 267.6 per 1,000, and no polypharmacy, 42.1 per 1,000). Additionally, pain medications were the most frequently ordered medication class overall, as well as in patients who exhibited major polypharmacy (477.3 per 1,000 patient visits), followed by CNS medications (185.8 per 1,000), and cardiovascular agents (182.4 per 1,000). In patients exhibiting moderate polypharmacy, CNS medications were the most frequently prescribed (97.6 per 1,000), followed by cardiovascular agents (85.7 per 1,000) and other high-risk medications (44.7 per 1,000). Like major polypharmacy, pain medications were the most frequently prescribed medication class in patients exhibiting minor polypharmacy (130.2 per 1,000), followed by CNS medications (64.8 per 1,000) and cardiovascular agents (29.9 per 1,000). Lastly, pain medications (16.8 per 1,000), CNS medications (9.1 per 1,000), and other medications (4.5 per 1,000) were the most frequently prescribed in patients with no polypharmacy (Fig 1).

**Fig 2:**
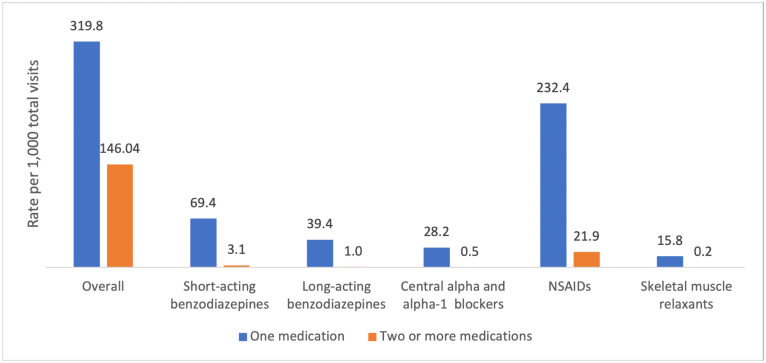
Rate of patients prescribed at least one high-risk medication overall and by medication class. ^+^ All comparisons between one medication and two or more medications were deemed statistically significant (p<0.0001).

For patients with any degree of polypharmacy, 319.8 per 1,000 total visits resulted in one high-risk medication. Additionally, 146.0 per 1,000 total visits resulted in two or more high-risk medications. Of all medication classes, high-risk non-steroidal anti-inflammatory drugs (NSAIDs) were the most frequently prescribed, with 232.4 per 1,000 total visits resulting in one high-risk NSAID, while 21.9 per 1,000 total visits resulted in two or more high-risk NSAIDs. Other high-risk medication classes, like short and long-acting benzodiazepines, central alpha blockers, alpha-1 blockers, and skeletal muscle relaxants, were also common (Fig 2).

## Discussion

In this nationally representative study of older Americans presenting to ambulatory physician offices in the U.S. between 2009 and 2016, a large proportion of visits resulted in some degree of polypharmacy. Importantly, there was a significant proportion of patients with polypharmacy that were prescribed high-risk medications. Overall, major polypharmacy (defined as patients more than five medications) was the most prevalent, accounting for over a third of U.S. physician office visits. This is one of the first studies to identify potential inappropriate or high-risk prescribing patterns in the older population.

Among the medication categories and classes prescribed to older patients in this study, the most common category and class were pain medications and NSAIDs, respectively. This is consistent with general prescribing trends, as the prevalence of NSAID prescribing in patients over 60 years of age was shown to be over 95% in the general practice setting [14]. While NSAIDs are known to be effective in treating pain and inflammation, they can result in several ADEs, namely nephrotoxicity, cardiovascular events, and GI ulceration. In addition, NSAIDs are a common cause of DDIs. Medications including antiplatelets, corticosteroids, selective serotonin reuptake inhibitors (SSRIs), and anticoagulants can all increase GI bleeding risk if taken with NSAIDs, especially when taken with multiple other medications or used chronically [15]. In addition to NSAIDs, skeletal muscle relaxants were also prevalent in this study. These medications, including carisoprodol and cyclobenzaprine, may increase fall risk in older patients due to their mechanism of action within the CNS. A study by Spence et al showed that risk of injury caused by skeletal muscle relaxant use in patients over 65 years increased compared to no use (OR 1.32, 95% CI 1.16–1.50, p<0.001) [16].

In addition to NSAIDs, there were also large proportions of older patients that were prescribed high-risk CNS medications (i.e., selective serotonin reuptake inhibitors, benzodiazepines, etc.). Overall, CNS medications have been associated with an increased risk of falls, hip fractures, and cognitive decline. For example, a study by Wright et al showed that CNS-active medication use was associated with cognitive decline (HR 1.27, 95% CI 0.90–1.81). [17] Both short and long-acting benzodiazepine use has also been known to show more risk than benefit in the elderly population. Withdrawal symptoms are prevalent in patients who have been exposed to a benzodiazepine, with approximately half of patients experiencing these symptoms [18]. As a result, both providers and patients have shown hesitancy in discontinuing benzodiazepines [19]. In addition to withdrawal symptoms, a study by Billioti de Gage et al showed that, after adjusting for other neurologic conditions like anxiety, depression, and insomnia, benzodiazepine use was associated with increased risk for the development of Alzheimer’s disease (aOR 1.43, 95% CI 1.28–1.60) [20].

Medication safety is an important factor that must be considered when treating the older population, particularly those vulnerable to polypharmacy. This responsibility is particularly important in pharmacy, whose role has greatly expanded from medication distribution to medication reconciliation and MTM, which aims to not only educate patients on their medications, but to also optimize their medication regimens through improving patient adherence and detecting potential ADEs and DDIs [21]. In addition, medication education is also important to the healthcare team, where pharmacists can aid in decreasing the number of inappropriate medications prescribed and subsequently, medication burden [21, 22]. Studies have shown that pharmacist intervention can significantly reduce the use of high-risk medications in the older population as categorized by the Beers Criteria^®^. For example, Chivapricha et al. compared the prevalence of PIMs (identified by the 2012 Beer’s Criteria^®^) upon hospital admission and discharge when adding a geriatric pharmacy specialist alongside the ward pharmacist in the inpatient setting. The study found that upon discharge, the prevalence of PIMs decreased significantly from admission (31.0% when the geriatric pharmacy specialist was with the ward pharmacist and 43.3% when the team only consisted of the ward pharmacist, p<0.05) [22].

Pharmacists are not the only healthcare professionals who play a critical role in polypharmacy. In a survey of multifaceted healthcare providers, the findings showed that physicians, pharmacists, and nurses all agreed that preventing polypharmacy was a role they all were responsible for [23]. For example, nursing-led interventions have shown an improved adherence rate to oral (85% to 90%) and inhaled treatment (37 to 71%) in COPD patients [23]. There are multiple screening tools for physicians to be more mindful of the possibility of adverse effects to PIMs [24]. Furthermore, in a study with nurse practitioners combatting polypharmacy through medication reviews, the importance of an interprofessional team regarding elderly care was highlighted [25]. However, further studies are still warranted to determine the optimal method to decrease the prevalence of polypharmacy.

Polypharmacy can occur due to many reasons; one simply being aging. As age-related changes occur, it can reduce hepatic blood flow and change drug clearance levels. Changes can also result from chronic diseases or malnutrition. Other causes include a lack of communication within the healthcare team, self-medicating without an accurate understanding of effects, or patients visiting multiple health systems that are not aware of what the other is doing [26].

Lastly, while there are several explanations as to why polypharmacy exists in this vulnerable patient population, it is also imperative to note that due to the degree of multi-morbid conditions, there are also instances where polypharmacy is unavoidable [26]. However, it has been noted that patients struggle with adherence in these situations. As a result, the utilization of medication organizers, electronic patient reminders about medication adherence, and combination medications can help alleviate medication burden [27]. With the collaborative effort of the patient’s healthcare team and the tools outlined above, adherence and safety can be assessed and potentially improved in those with high medication burden.

### Limitations

Finally, there are some potential limitations in this study. As the NAMCS dataset provides information from single office visits, previous visits and longitudinal follow-ups were unavailable. However, as the NAMCS includes a random sample of visits from various physician offices in the country, there is a low probability that one patient was sampled multiple times. In addition, medication data from this dataset only indicate which medications patients were newly prescribed or were taking at the time of visit with or without the corresponding disease state for which it was being prescribed. Therefore, this study is unable to account for the accuracy of this list in terms of previous medications, active medications, or chronic use of these medications. As such, this study was not able to concretely differentiate between essential and inappropriate polypharmacy, but it did identify potentially inappropriate medications based on the drug class and the age of the population studied. Next, the survey used in this study collected data on only outpatient physician offices, so study findings are not representative and can underestimate high-risk medication prescribing in the elderly in the U.S., particularly in the inpatient setting and over-the-counter medications. Lastly, due to the survey setting, this study was also unable to account for patients getting medications filled from multiple physicians or picking up medications from multiple pharmacies.

## Conclusion

In this nationally representative study, polypharmacy and more specifically, major polypharmacy, was prevalent in U.S. physician offices within the elderly population. High-risk medications were also common in this population, with high-risk pain medications being the most commonly prescribed. Findings from this study support enhanced pharmacist roles in medication therapy management in order to improve drug therapy regimens in the elderly population.

## Supporting information

S1 Table Beer’s criteria medication list and Multum codes. (DOCX)

## References

[1] Waite LJ. The Demographic Faces of the Elderly. Popul Dev Rev. 2004;30(Supplement):3–16. 19129925 PMC2614322

[2] Maher RL, Hanlon J, Hajjar ER. Clinical consequences of polypharmacy in elderly. Expert Opin Drug Saf. 2014;13(1):57–65. doi: 10.1517/14740338.2013.827660 24073682 PMC3864987

[3] Hudhra K, Garcia-Caballos M, Casado-Fernandez E, Jucja B, Shabani D, Bueno-Cavanillas A. Polypharmacy and potentially inappropriate prescriptions identified by Beers and STOPP criteria in co-morbid older patients at hospital discharge. J Eval Clin Pract. 2016;22(2):189–93. doi: 10.1111/jep.12452 26399173

[4] Zhang N, Sundquist J, Sundquist K, Ji J. An Increasing Trend in the Prevalence of Polypharmacy in Sweden: A Nationwide Register-Based Study. Front Pharmacol. 2020;11:326. doi: 10.3389/fphar.2020.00326 32265705 PMC7103636

[5] Gurwitz JH, Field TS, Harrold LR, Rothschild J, Debellis K, Seger AC, et al. Incidence and preventability of adverse drug events among older persons in the ambulatory setting. JAMA. 2003;289(9):1107–16. doi: 10.1001/jama.289.9.1107 12622580

[6] Oscanoa TJ, Lizaraso F, Carvajal A. Hospital admissions due to adverse drug reactions in the elderly. A meta-analysis. Eur J Clin Pharmacol. 2017;73(6):759–70. doi: 10.1007/s00228-017-2225-3 28251277

[7] Chan M, Nicklason F, Vial JH. Adverse drug events as a cause of hospital admission in the elderly. Intern Med J. 2001;31(4):199–205. doi: 10.1046/j.1445-5994.2001.00044.x 11456032

[8] Doan J, Zakrzewski-Jakubiak H, Roy J, Turgeon J, Tannenbaum C. Prevalence and risk of potential cytochrome P450-mediated drug-drug interactions in older hospitalized patients with polypharmacy. Ann Pharmacother. 2013;47(3):324–32. doi: 10.1345/aph.1R621 23482734

[9] Juurlink DN, Mamdani M, Kopp A, Laupacis A, Redelmeier DA. Drug-drug interactions among elderly patients hospitalized for drug toxicity. JAMA. 2003;289(13):1652–8. doi: 10.1001/jama.289.13.1652 12672733

[10] Zhao Y, Wong FK. Effects of a postdischarge transitional care programme for patients with coronary heart disease in China: a randomised controlled trial. J Clin Nurs. 2009;18(17):2444–55. doi: 10.1111/j.1365-2702.2009.02835.x 19619203

[11] By the American Geriatrics Society Beers Criteria Update Expert P. American Geriatrics Society 2019 Updated AGS Beers Criteria(R) for Potentially Inappropriate Medication Use in Older Adults. J Am Geriatr Soc. 2019;67(4):674–94. doi: 10.1111/jgs.15767 30693946

[12] O’Mahony D, O’Sullivan D, Byrne S, O’Connor MN, Ryan C, Gallagher P. STOPP/START criteria for potentially inappropriate prescribing in older people: version 2. Age Ageing. 2015;44(2):213–8. doi: 10.1093/ageing/afu145 25324330 PMC4339726

[13] Claxton AJ, Cramer J, Pierce C. A systematic review of the associations between dose regimens and medication compliance. Clin Ther. 2001;23(8):1296–310. doi: 10.1016/s0149-2918(01)80109-0 11558866

[14] Pilotto A, Franceschi M, Leandro G, Di Mario F, Geriatric Gastroenterology Study G. NSAID and aspirin use by the elderly in general practice: effect on gastrointestinal symptoms and therapies. Drugs Aging. 2003;20(9):701–10. doi: 10.2165/00002512-200320090-00006 12831293

[15] Wongrakpanich S, Wongrakpanich A, Melhado K, Rangaswami J. A Comprehensive Review of Non-Steroidal Anti-Inflammatory Drug Use in The Elderly. Aging Dis. 2018;9(1):143–50. doi: 10.14336/AD.2017.0306 29392089 PMC5772852

[16] Spence MM, Shin PJ, Lee EA, Gibbs NE. Risk of injury associated with skeletal muscle relaxant use in older adults. Ann Pharmacother. 2013;47(7–8):993–8. doi: 10.1345/aph.1R735 23821610

[17] Wright RM, Roumani YF, Boudreau R, Newman AB, Ruby CM, Studenski SA, et al. Effect of central nervous system medication use on decline in cognition in community-dwelling older adults: findings from the Health, Aging And Body Composition Study. J Am Geriatr Soc. 2009;57(2):243–50. doi: 10.1111/j.1532-5415.2008.02127.x 19207141 PMC2744424

[18] Vicens C, Bejarano F, Sempere E, Mateu C, Fiol F, Socias I, et al. Comparative efficacy of two interventions to discontinue long-term benzodiazepine use: cluster randomised controlled trial in primary care. Br J Psychiatry. 2014;204(6):471–9. doi: 10.1192/bjp.bp.113.134650 24526745

[19] Tannenbaum C, Martin P, Tamblyn R, Benedetti A, Ahmed S. Reduction of inappropriate benzodiazepine prescriptions among older adults through direct patient education: the EMPOWER cluster randomized trial. JAMA Intern Med. 2014;174(6):890–8. doi: 10.1001/jamainternmed.2014.949 24733354

[20] Billioti de Gage S, Moride Y, Ducruet T, Kurth T, Verdoux H, Tournier M, et al. Benzodiazepine use and risk of Alzheimer’s disease: case-control study. BMJ. 2014;349:g5205. doi: 10.1136/bmj.g5205 25208536 PMC4159609

[21] Weddle SC, Rowe AS, Jeter JW, Renwick RC, Chamberlin SM, Franks AS. Assessment of Clinical Pharmacy Interventions to Reduce Outpatient Use of High-Risk Medications in the Elderly. J Manag Care Spec Pharm. 2017;23(5):520–4. doi: 10.18553/jmcp.2017.23.5.520 28448781 PMC10397936

[22] Chivapricha W, Srinonprasert V, Suansanae T. Impact of Geriatric Pharmacy Specialist Interventions to Reduce Potentially Inappropriate Medication Among Hospitalized Elderly Patients at Medical Wards: A Prospective Quasi-Experimental Study. Drugs Real World Outcomes. 2020. doi: 10.1007/s40801-020-00214-7 33063296 PMC7984164

[23] Farrell B, Thompson W, Black CD, Archibald D, Raman-Wilms L, Grassau P, et al. Health care providers’ roles and responsibilities in management of polypharmacy: Results of a modified Delphi. Can Pharm J (Ott). 2018;151(6):395–407. doi: 10.1177/1715163518804276 30559915 PMC6293398

[24] Baruth JM, Gentry MT, Rummans TA, Miller DM, Burton MC. Polypharmacy in older adults: the role of the multidisciplinary team. Hosp Pract (1995). 2020;48(sup1):56–62. doi: 10.1080/21548331.2019.1706995 31900000

[25] Bergman-Evans B. A nurse practitioner led protocol to address polypharmacy in long-term care. Geriatr Nurs. 2020;41(6):956–61. doi: 10.1016/j.gerinurse.2020.07.002 32718755 PMC7380258

[26] Dagli RJ, Sharma A. Polypharmacy: a global risk factor for elderly people. J Int Oral Health. 2014;6(6):i–ii. 25628499 PMC4295469

[27] Kini V, Ho PM. Interventions to Improve Medication Adherence: A Review. JAMA. 2018;320(23):2461–73. doi: 10.1001/jama.2018.19271 30561486

