# Supplementary material for: Polypharmacy prevalence in older adults seen in United States physician offices from 2009 to 2016
Source: PLoS One. 2021 Aug 3;16(8):e0255642. doi: 10.1371/journal.pone.0255642 (PMC8330900; doi:10.1371/journal.pone.0255642)
Supplement: S1 Table — (DOCX) [file pone.0255642.s001.docx]

| **Medication group** | **Medication class** | **Multum code(s)** |
| --- | --- | --- |
| **Anticholinergics** | **First generation antihistamines** |  |
|  | Brompheniramine | d00785 |
|  | Carbinoxamine | d03569 |
|  | Chlorpheniramine | d00191 |
|  | Clemastine | d00780 |
|  | Cyproheptadine | d00790 |
|  | Dexbrompheniramine | d03750 |
|  | Dexchlorpheniramine | d00784 |
|  | Diphenydramine | d00212 |
|  | Doxylamine | d03154 |
|  | Hydroxyzine | d00907 |
|  | Promethazine | d00787 |
|  | Triprolidine | d00786 |
|  | **Antiparkinson agents** |  |
|  | Benztropine | d00175 |
|  | Trihexyphenidyl | d00970 |
|  | **Antispasmodics** |  |
|  | Belladonna alkaloids | d03485 |
|  | Clidinium-chlordiazepoxide | d03492 |
|  | Dicyclomine | d00999 |
|  | Hyoscyamine | d00985 |
|  | Propantheline | d00359 |
|  | Scopolamine | d00986 |
|  | **Antithrombotics** |  |
|  | Dipyridamole | d00213 |
|  | Ticlopidine | d00514 |
| **Cardiovascular agents** | **Alpha-1 blockers** |  |
|  | Doxazosin | d00726 |
|  | Prazosin | d00138 |
|  | Terazosin | d00386 |
|  | **Central alpha blockers** |  |
|  | Clonidine | d00044 |
|  | Guanabenz | d00130 |
|  | Guanfacine | d00717 |
|  | Methyldopa | d00133 |
|  | Reserpine | d00367 |
|  | **Antiarrhythmic drugs (1a, 1c, III)** |  |
|  | Amiodarone | d00002 |
|  | Dofetilide | d04459 |
|  | Dronedarone | d07458 |
|  | Flecainide | d00234 |
|  | ibutilide | d03871 |
|  | Procainamide | d00075 |
|  | Propafenone | d00358 |
|  | Quinidine | d00020 |
|  | Sotalol | d00371 |
|  | **Other cardiovascular agents** |  |
|  | Disopyramide | d00214 |
|  | Dronedarone | d07458 |
|  | Digoxin | d00210 |
|  | Nifedipine, IR | d00051 |
|  | Spironolactone | d00373 |
| **CNS agents** | **Tertiary TCAs** |  |
|  | Amitriptyline | d00146 |
|  | Chlordiazepoxide-amitriptyline | d03462 |
|  | Clomipramine | d00876 |
|  | Doxepin | d00217 |
|  | Imipramine | d00259 |
|  | Perphenazine-amitriptyline | d03463 |
|  | Trimipramine | d00873 |
|  | **First and second generation antipsychotics** |  |
|  | Chlorpromazine | d00064 |
|  | Fluphenazine | d00237 |
|  | Haloperidol | d00027 |
|  | Loxapine | d00897 |
|  | Mesoridazine |  |
|  | Molindone | d00896 |
|  | Perphenazine | d00855 |
|  | Pimozide | d00898 |
|  | Promazine | d00356 |
|  | Thioridazine | d00389 |
|  | Thiothixene | d00391 |
|  | Trifluoperazine | d00890 |
|  | Aripiprazole | d04825 |
|  | Asenapine | d07473 |
|  | Clozapine | d00199 |
|  | Iloperidone | d07441 |
|  | Lurasidone | d07705 |
|  | Olanzapine | d04050 |
|  | Paliperidone | d06297 |
|  | Quetiapine | d04220 |
|  | Risperidone | d03180 |
|  | Ziprasidone | d04747 |
|  | **Barbituates** |  |
|  | Amobarbital | d00171 |
|  | Butabarbital | d00923 |
|  | Butalbital | d03061 |
|  | Mephobarbital | d00919 |
|  | Pentobarbital | d00335 |
|  | Phenobarbital | d00340 |
|  | Secobarbital | d00368 |
|  | **Benzodiazepines** |  |
|  | Alprazolam | d00168 |
|  | Estazolam | d00915 |
|  | Lorazepam | d00149 |
|  | Oxazepam | d00040 |
|  | Temazepam | d00384 |
|  | Triazolam | d00397 |
|  | Chlordiazepoxide | d00189 |
|  | Chlordiazepoxide-amitriptyline | d03462 |
|  | Clidinium-chlordiazepoxide | d03492 |
|  | Clonazepam | d00197 |
|  | Diazepam | d00148 |
|  | Flurazepam | d00238 |
|  | Quazepam | d00917 |
|  | **Nonbenzodiazepine hypnotics** |  |
|  | Eszopiclone | d05421 |
|  | Zolpidem | d00910 |
|  | Zaleplon | d00910 |
|  | **Other CNS agents** |  |
|  | Chloral hydrate  Meprobamate | d00147  d00288 |
|  | Ergot mesylates | d00902 |
| **Pain medications** | **NSAIDs** |  |
|  | Aspirin | d00170 |
|  | Diclofenac | d00848 |
|  | Diflunisal | d00208 |
|  | Etodolac | d00851 |
|  | Fenoprofen | d00026 |
|  | Ibuprofen | d00015 |
|  | Indomethacin | d00039 |
|  | Ketorolac | d00273 |
|  | Ketoprofen | d00028 |
|  | Meclofenamate | d00283 |
|  | Mefenamic acid | d00285 |
|  | Meloxicam | d04532 |
|  | Nabumetone | d00310 |
|  | Naproxen | d00019 |
|  | Oxaprozin | d00853 |
|  | Piroxicam | d00343 |
|  | Sulindac | d00033 |
|  | Tolmetin | d00054 |
|  | **Skeletal muscle relaxants** |  |
|  | Carisoprodol | d00960 |
|  | Cyclobenzaprine | d00963 |
|  | Metaxalone | d00964 |
|  | Methocarbamol | d00965 |
|  | Orphenadrine | d00966 |
|  | **Other pain medications** |  |
|  | Pentazocine | d00334 |
| **Other medications** |  |  |
|  | **Anti-infectives** |  |
|  | Nitrofurantoin | d00112 |
|  | **Endocrine medications** |  |
|  | Methyltestosterone | d00295 |
|  | Testosterone | d00558 |
|  | Desiccated thyroid | d00655 |
|  | Estrogens (with or without progestins) | a11518 |
|  | Conjugated estrogens | d00541 |
|  | Progesterone | d00550 |
|  | Growth hormone | c00100 |
|  | Insulin | d00262 |
|  | Megestrol | d01348 |
|  | Chlorpropamide | d00042 |
|  | Glyburide | d00248 |
|  | **Gastrointestinal medications** |  |
|  | Metoclopramide | d00298 |
|  | Mineral oil | d01019 |
|  | Trimethobenzamide | d00862 |
